# The Effect of Ethnic Variation on the Success of Induced Labour in Nulliparous Women with Postdates Pregnancies

**DOI:** 10.1155/2016/9569725

**Published:** 2016-02-24

**Authors:** Dimitrios Papoutsis, Angeliki Antonakou, Chara Tzavara

**Affiliations:** ^1^Department of Obstetrics and Gynaecology, Shrewsbury and Telford Hospital NHS Trust, Telford TF1 6FT, UK; ^2^Department of Midwifery, Midwifery School, Alexander Technological Educational Institute of Thessaloniki, 57 400 Thessaloniki, Greece; ^3^Department of Hygiene, Epidemiology and Medical Statistics, Medical School, University of Athens, 115 27 Athens, Greece

## Abstract

*Objective*. To identify the potential effect of ethnic variation on the success of induction of labour in nulliparous women with postdates pregnancies.* Study Design*. This was an observational cohort study of women being induced for postdates pregnancies (≥41 weeks) between 2007 and 2013. Women induced for stillbirths and with multiple pregnancies were excluded. The primary objective was to identify the effect of ethnicity on the caesarean section (CS) delivery rates in this cohort of women.* Results*. 1,636 nulliparous women were identified with a mean age of 27.2 years. 95.8% of the women were of White ethnic origin, 2.6% were Asian, and 1.6% were of Black ethnic origin. The CS delivery rate was 24.4% in the total sample. Women of Black ethnic origin had a 3.26 times greater likelihood for CS in comparison to White women, after adjusting for maternal age, BMI, smoking, presence of meconium, use of epidural analgesia, fetal gender, birth weight, and head circumference (adjusted OR = 3.26; 95% CI: 1.31–8.08,* p* = 0.011).* Conclusion*. We have found that nulliparous women of Black ethnicity demonstrate an almost threefold increased risk of caesarean section delivery when induced for postdates pregnancy.

## 1. Introduction

Postterm pregnancy has been defined as a pregnancy extending at or beyond 42 weeks of gestation [1] and has a reported incidence of 7% in all pregnancies [2]. Despite the improved understanding of the complex interplay between hormonal, mechanical, and inflammatory processes that initiate labour and allow its progression, the pathogenesis of postterm pregnancy still remains unclear [3]. The National Institute for Health and Care Excellence (NICE) in the United Kingdom has recommended that women over 41 weeks of gestation should have labour induced for the prevention of stillbirth due to postmaturity [4]. A Cochrane review has also reported that inducing labour at 41 weeks of gestation does not increase the caesarean section (CS) rates and leads to improved perinatal outcomes [5]. However, the NICE recommendation in 2008 does not take into account the significant influence of ethnic variation on the obstetric and perinatal outcome [6, 7]. There is evidence that normal gestational length is shorter by a week in Black and Asian women when compared with White European women and that fetal maturation occurs earlier [8]. Moreover, the incidence of stillbirth after 41 weeks of gestation is reported to be higher for African and Indian women as compared to Caucasian women [9, 10].

Since the national guidelines have not yet been adjusted to include ethnic origin, we sought to determine with our study the potential effect of ethnic variation on the success rates of inducing labour in a cohort of nulliparous women with postdates pregnancies.

## 2. Materials and Methods

This was an observational cohort study of women induced for postdates pregnancies (gestational age ≥41 weeks) at the Maternity Unit of the Shrewsbury and Telford Hospital (SaTH) National Health Service (NHS) Trust, between January 2007 and December 2013. Nulliparous women with singleton cephalic presentation deliveries were considered eligible for the study. Women induced for stillbirths and fetal congenital abnormalities and women with multiple pregnancies were excluded. Data was collected from Medway® obstetric electronic database and maternal data, labour/delivery data, and neonatal data were all recorded.

Maternal data recorded were age, body mass index (BMI) at booking, smoking status, and ethnicity. Labour and delivery data included route of birth (vaginal delivery, caesarean section delivery), epidural analgesia use, and liquor appearance (normal, meconium stained). Neonatal data recorded were fetal gender (male, female), birth weight, head circumference, Apgar scores (at 1 and 5 minutes), umbilical cord gases taken at delivery (arterial, venous), and admission to the neonatal unit (NNU).

Ethnicity data were self-reported during the first antenatal visit of women and the cohort of White European women was considered the reference group. Black African and Black Caribbean women were combined into the group of women with Black ethnic origin. Women from India or Pakistan or those reported from Asia were combined into the group of women with Asian ethnic origin. Women who had not stated their ethnicity (*n* = 93 or 5% of the sample) at booking were excluded from the purposes of the study.

Quantitative variables were expressed as mean values (SD: standard deviation) and qualitative variables were expressed as absolute and relative frequencies. For the comparison of proportions, chi-square and Fisher's exact tests were used. Analysis of variance (ANOVA) and Student's *t*-test were performed for the comparison of mean values between the ethnic groups. Bonferroni correction was used in order to control for type I error in the case of multiple comparisons. Logistic regression analysis was used in order to determine independent factors that were associated with the likelihood of caesarean section delivery. Adjusted odds ratios (OR) with 95% confidence intervals (95% CI) were computed from the results of the logistic regression analyses. All reported *p* values were two-tailed. Statistical significance was set at *p* < 0.05 and analyses were conducted using SPSS statistical software (version 20.0).

Ethical approval for collection and analysis of data in our study was obtained by the Research and Development Department of the Shrewsbury and Telford Hospital NHS Trust.

## 3. Results

The total sample consisted of 1,636 eligible women with a mean maternal age at delivery of 27.2 years (SD = 6.2 years). 95.8% of the women were of White ethnic origin, 2.6% were Asian, and 1.6% were of Black ethnic origin. The mean value of BMI was 26.7 kg/m^2^ (SD = 6.2 kg/m^2^) and more than half of the participants (57.0%) never smoked. During labour, 33.7% of the women used epidural analgesia for pain relief and the overall caesarean section delivery rate was 24.4%. The mean birth weight was 3709.2 gr (SD = 430.8 gr) with 52.3% of the fetuses being male. Meconium stained liquor appearance was identified in 21.2% of the participants and 4.2% of the newborns were admitted to the neonatal unit (Table 1).

The participants' characteristics for each ethnic category are presented in Table 2. The proportion of caesarean sections was 23.9% in White women, 28.6% in Asian women, and 46.2% in Black women, and it was significantly greater in Black women as compared to White women (*p* = 0.009). We also found that Black women when compared to White women had a trend for a slightly shorter gestational length (291.1 ± 2.2 days versus 291.9 ± 2.2 days, *p* = 0.07).

Multiple logistic regression analysis with dependent variable, the CS delivery rate, showed that ethnicity was independently associated with the odds for CS delivery (Table 3). Specifically, Black women had a 3.26 times greater likelihood for caesarean section in comparison to White women after adjusting for mother's age, BMI, smoking status, presence of meconium, use of an epidural, fetal gender, birth weight, and head circumference. Other factors that were also found to be independently associated with a greater likelihood for caesarean delivery were maternal age, maternal BMI, the presence of meconium, the use of epidural analgesia, and the increased birth weight and head circumference.

## 4. Discussion

Our study has shown that the overall CS delivery rate in our cohort of nulliparous women who were induced for postdates pregnancy was 24.4%. This is near the national average in the United Kingdom where the mean emergency CS rate for 2011-2012 was 30.2% in primiparous and 13.2% in multiparous women whose labours were induced [11, 12]. Subgroup analysis of our data demonstrated that women of Black ethnic origin had an almost threefold increased risk of caesarean section delivery in comparison to White women after adjusting for age, BMI, smoking, presence of meconium, use of an epidural, fetal gender, birth weight, and head circumference. Nevertheless, no significant differences were noted in neonatal outcomes between the three ethnic groups (White European, Black, and Asian) in terms of Apgar scores, umbilical cord gases, or admissions to the neonatal unit.

There are several studies reporting on the length of gestation amongst different ethnic groups. A large population-based study showed that the median gestational length was 39 completed weeks in Black and Asian women in comparison to 40 completed weeks in White European women [8]. Another study has shown that gestational duration is more strongly associated with the mother's rather than the father's ethnic origin [13]. Ethnic differences have also been noted with preterm delivery rates where gestation was reported to be shorter for UK African and Afro-Caribbean women when compared to Caucasian women even after adjusting for confounding factors [14]. There is also evidence that Afro-Caribbean women have a twofold higher rate of stillbirths in all maternal age groups compared to Caucasian and Asian ethnicity, even when adjusted for parity and medical comorbidities [15, 16]. Moreover, the incidence of stillbirth after 41 weeks of gestation has been reported to be higher for African women when compared to Caucasian women [10, 17]. Ethnic disparities have also been reported regarding the perinatal mortality rates at 40 and 41 weeks of gestation with an almost twofold increased perinatal mortality rate for Black women when compared to White Europeans [17].

The hypothesis that has been stipulated to explain the shorter gestational length in spontaneous onset labours, the higher preterm delivery rates, the higher perinatal mortality, and stillbirth rates in the case of Black women is the earlier maturation of infants delivered within this ethnic group [8]. This hypothesis explains why the perinatal mortality rates in Black-ethnicity infants are reported to be lower than their White-ethnicity counterparts in the case of preterm delivery [18]. It may also explain why Black infants born at term have higher perinatal mortality rates compared to White infants, since they may be facing the complications of postmaturity at earlier gestation than the White infants [8, 19]. Other evidence for the hypothesis of earlier fetal maturation is that Black infants both at term and at preterm are more likely than White infants to pass meconium* in utero*, which is considered an indication of maturity [8, 20]. In our study, we found similar rates of meconium passage and neonatal outcomes amongst the different ethnic subgroups. Perhaps our study was underpowered to detect any significant differences on this outcome. What our study has shown however in support of the theory of earlier maturation for Black infants is that there is a trend (*p* = 0.07) for a slightly shorter gestational length in Black women when compared to White women in our cohort.

As this was a retrospective analysis of a large set of data, there are certain weaknesses to be considered. First, all information was collected from an electronic database where accuracy of the data is dependent on the practitioner inputting the information. Second, ethnicity was self-reported at booking and this may have led to misclassification. Third, there was a subgroup of women in our cohort with missing data on ethnicity (*n* = 93 or 5% of the sample). This may reflect either noncollected data or women unwilling to disclose their ethnicity. If this number was classified into a specific ethnic group, then perhaps results may have been different. Fourth, on the basis of possible earlier maturation of Black and Asian fetuses, a higher proportion of women with Black and Asian ethnic origin when compared to White European origin may have gone into spontaneous labour much earlier and prior to the induction of labour date at 41 weeks of gestation. This means that the subgroups of Black and Asian women may be underrepresented in our cohort of women with induced labour and this may have introduced a bias in our results. The main strength of our study includes its large sample size with inclusion of nulliparous only women so as to account for the significant confounding effect of parity.

In conclusion, we have found that nulliparous women of Black ethnicity demonstrate an almost threefold increased risk of caesarean section delivery when induced for postdates pregnancy at 41 weeks of gestation. Our study lends support to the literature reports that ethnic differences are likely to play a role in postdates pregnancy. As there is evidence that babies of different ethnic origin most likely mature at different rates, it remains to be seen in future studies whether an earlier induction policy in certain ethnic groups may reduce the rates of caesarean delivery in these women.

## Figures and Tables

**Table 1:** Total sample characteristics (*n* = 1,636).

	*N* (%)
Mother's age at delivery (years), mean (SD)	27.2 (6.2)
Ethnicity	
White	1568 (95.8)
Black	26 (1.6)
Asian	42 (2.6)
BMI (kg/m^2^), mean (SD)	26.7 (5.7)
Smoking	
Nonsmoker (never)	911 (57.0)
Current smoker	169 (10.6)
Previous smoker	519 (32.5)
Presence of meconium	
No	1285 (78.8)
Yes	345 (21.2)
Use of an epidural	
No	1085 (66.3)
Yes	551 (33.7)
Route of birth	
Caesarean section delivery	399 (24.4)
Vaginal route	1237 (75.6)
Normal vaginal delivery	1073 (86.7%)
Instrumental delivery	164 (13.2%)
Fetal gender	
Female	781 (47.7)
Male	855 (52.3)
Birth weight, mean (SD)	3709.2 (430.8)
Head circumference at birth (cm), mean (SD)	35.5 (1.4)
Apgar score at 1 minute, mean (SD)	8.3 (1.6)
Apgar score at 5 minutes, mean (SD)	9.6 (0.9)
Cord gases taken at delivery, arterial pH, mean (SD)	7.23 (0.08)
Cord gases taken at delivery, venous pH, mean (SD)	7.28 (0.07)
Admitted to NNU	
No	1319 (95.8)
Yes	58 (4.2)

**Table 2:** Demographics and perinatal characteristics by ethnic categories.

	Ethnicity			*p*
	White	Black	Asian	
	*N* (%)	*N* (%)	*N* (%)	
Mother's age at delivery (years), mean (SD)	27.2 (6.3)	26.4 (5.3)	28.1 (4.4)	0.583^*∗*^
Gestational age at delivery (days), mean (SD)	291.9 (2.2)	291.1 (2.2)	292.1 (2.7)	0.183^*∗*^
BMI (kg/m^2^), mean (SD)	26.7 (5.7)	26.5 (5.2)	24.9 (5.1)	0.187^*∗*^
Smoking				
Nonsmoker (never)	856 (55.9)	20 (76.9)	35 (85.4)	<0.001^++^
Current smoker	167 (10.9)	2 (7.7)	0 (0.0)	
Previous smoker	509 (33.2)	4 (15.4)	6 (14.6)	
Presence of meconium				
No	1233 (78.9)	20 (76.9)	32 (76.2)	0.886^+^
Yes	329 (21.1)	6 (23.1)	10 (23.8)	
Use of an epidural				
No	1036 (66.1)	22 (84.6)	27 (64.3)	0.134^+^
Yes	532 (33.9)	4 (15.4)	15 (35.7)	
Route of birth				
Caesarean section delivery	375 (23.9)	12 (46.2)	12 (28.6)	0.026^+^
Vaginal route	1193 (76.1)	14 (53.8)	30 (71.4)	
Fetal gender				
Female	752 (48)	8 (30.8)	21 (50.0)	0.210^+^
Male	816 (52)	18 (69.2)	21 (50.0)	
Birth weight, mean (SD)	3713.4 (430.1)	3723.1 (428.1)	3542.3 (436.4)	0.039^*∗*^
Head circumference at birth (cm), mean (SD)	35.5 (1.4)	35.4 (1.2)	34.9 (1.2)	0.012^*∗*^
Apgar score at 1 minute, mean (SD)	8.3 (1.6)	8 (1.5)	8.3 (1.4)	0.573^*∗*^
Apgar score at 5 minutes, mean (SD)	9.6 (0.9)	9.4 (0.8)	9.5 (0.7)	0.365^*∗*^
Cord gases at delivery, arterial pH, mean (SD)	7.23 (0.08)	7.22 (0.09)	7.21 (0.08)	0.414^*∗*^
Cord gases at delivery, venous pH, mean (SD)	7.25 (0.07)	7.25 (0.09)	7.25 (0.08)	0.114^*∗*^
Admitted to NNU				
No	1265 (95.8)	22 (100.0)	32 (94.1)	0.735^++^
Yes	56 (4.2)	0 (0.0)	2 (5.9)	

^*∗*^ANOVA; ^+^Pearson's chi-square test; ^++^Fisher's exact test.

**Table 3:** Odds ratios (OR) and 95% confidence intervals (CI) derived from multiple logistic regression analysis for caesarean section in relation to demographic and perinatal variables.

	OR (95% CI)	*p*
Mother's age at delivery (years)	1.04 (1.02–1.06)	0.001
Ethnicity		
White	1.00^‡^	
Black	3.26 (1.31–8.08)	0.011
Asian	2.07 (0.95–4.51)	0.066
BMI (kg/m^2^)	1.03 (1–1.05)	0.026
Smoking		
Nonsmoker (never)	1.00	
Current smoker	0.86 (0.51–1.47)	0.590
Previous smoker	0.86 (0.64–1.16)	0.320
Presence of meconium	1.98 (1.47–2.69)	<0.001
Use of an epidural	2.28 (1.73–2.99)	<0.001
Fetal gender		
Female	1.00	
Male	1.04 (0.78–1.37)	0.808
Birth weight (per 100 gr increase)	1.07 (1.03–1.11)	0.001
Head circumference at birth (per 1 cm increase)	1.23 (1.12–1.36)	<0.001

^‡^Reference category.
